# The LORIS MyeliNeuroGene rare disease database for natural history studies and clinical trial readiness

**DOI:** 10.1186/s13023-021-01953-8

**Published:** 2021-07-23

**Authors:** Aaron Spahr, Zaliqa Rosli, Mélanie Legault, Luan T. Tran, Simon Fournier, Helia Toutounchi, Lama Darbelli, Cécile Madjar, Cassandra Lucia, Marie-Lou St-Jean, Samir Das, Alan C. Evans, Geneviève Bernard

**Affiliations:** 1 Department of Neurology and Neurosurgery, McGill University, Montréal, Québec Canada; 2 Department of Pediatrics, McGill University, Montréal, Québec Canada; 3 Department of Human Genetics, McGill University, Montréal, Québec Canada; 4 Department of Specialized Medicine, Division of Medical Genetics, McGill University Health Centre, Montréal, Québec Canada; 5 Child Health and Human Development Program, Research Institute, McGill University Health Center, Montréal, Québec Canada; 6 McGill Centre for Integrative Neuroscience, Montreal Neurological Institute, McGill University, Montréal, Québec Canada

**Keywords:** Leukodystrophy, Rare diseases, Information management systems, Databases, Registry, Natural history, Outcome measures, Clinical trials, Biomarkers

## Abstract

**Background:**

Rare diseases are estimated to affect 150–350 million people worldwide. With advances in next generation sequencing, the number of known disease-causing genes has increased significantly, opening the door for therapy development. Rare disease research has therefore pivoted from gene discovery to the exploration of potential therapies. With impending clinical trials on the horizon, researchers are in urgent need of natural history studies to help them identify surrogate markers, validate outcome measures, define historical control patients, and design therapeutic trials.

**Results:**

We customized a browser-accessible multi-modal (e.g. genetics, imaging, behavioral, patient-determined outcomes) database to increase cohort sizes, identify surrogate markers, and foster international collaborations. Ninety data entry forms were developed including family, perinatal, developmental history, clinical examinations, diagnostic investigations, neurological evaluations (i.e. spasticity, dystonia, ataxia, etc.), disability measures, parental stress, and quality of life. A customizable clinical letter generator was created to assist in continuity of patient care.

**Conclusions:**

Small cohorts and underpowered studies are a major challenge for rare disease research. This online, rare disease database will be accessible from all over the world, making it easier to share and disseminate data. We have outlined the methodology to become Title 21 Code of Federal Regulations Part 11 Compliant, which is a requirement to use electronic records as historical controls in clinical trials in the United States. Food and Drug Administration compliant databases will be life-changing for patients and families when historical control data is used for emerging clinical trials. Future work will leverage these tools to delineate the natural history of several rare diseases and we are confident that this database will be used on a larger scale to improve care for patients affected with rare diseases.

## Background

According to the World Health Organization (WHO), the definition of a rare disease is one that affects every 1 in 2000 people or less. The global prevalence of these approximately 8000 rare genetic disorders is estimated to be between 150 and 350 million people [1–7]. Historically, rare diseases have been notoriously difficult to diagnose due to their heterogeneous phenotypes and genotypes [8]. Since only around 5% of all rare diseases have a FDA-approved treatment, many orphan diseases utilize off-label indications of medications approved for other purposes [9]. However, an incredible amount of advancement in the description of novel rare disease entities and the identification of novel disease-causing genes has been accomplished over the last decade using rapidly evolving genetic technologies, including with the most recent use of next generation sequencing (NGS). Opening the door for studies investigating disease pathogenesis and potential therapeutic approaches has pivoted rare disease research from gene discovery towards investigating potential treatments [6]

With impending clinical trials on the horizon, rare disease researchers are realizing a tremendous need for natural history data [10, 11]. The goal of a natural history study is to recruit patients for longitudinal analysis of natural disease progression [12]. The data gathered is used to help identify surrogate markers, determine the best outcome measures to be used in potential therapeutic trials, can serve as the control arm and serve as benchmarks for efficacy in one arm rare disease trials [13–17]. Natural history studies result in incredible amounts of information being collected, including clinical, behavioral, sociodemographic, genetic, imaging, and patient and family reported outcomes.

This diversity and quantity of data can be difficult to manage, so rare disease researchers must begin to utilize information management systems, or databases, to facilitate natural history studies. Rare disease research relies heavily on international collaboration and data sharing in order to recruit large patient populations to obtain adequate statistical power [6, 18]. Therefore, utilizing an online database can uniquely benefit rare disease research more than other disease research fields where significant patient populations are more prevalent [19].

If rare disease databases are going to be successful in future clinical trials, they must adhere to local and international regulations for electronic records. Title 21 Code of Federal Regulations (CFR) Part 11 published in 1997, from the U.S. Food and Drug Administration, outlines what is considered trustworthy, reliable record keeping. These regulations apply to any FDA-regulated industry, such as pharmaceutical companies, medical device manufacturers, biotechnological companies, and clinical research organizations. We chose to adhere to all general requirements that will be detailed below in the Methods section.

There are a variety of different databases available to aid researchers such as RedCap [20], Deduce [21], HID [22], DFBIdb [23], LONI [24], MIND [25], NeuroLOG [26], etc. We elected to customize the Longitudinal Online Research and Imaging System (LORIS) [27–30] to help organize data and facilitate international collaborations when conducting multi-site natural history studies because of its strong track record and the fact that it is open source. Here, we detail below how our group used LORIS and 21 CFR Part 11 guidelines to set up workflows and develop the LORIS MyeliNeuroGene Database for Rare Diseases to lead us to clinical trial preparedness in the coming years.

## Results

An instance of LORIS was installed and configured for the MyeliNeuroGene Research Group at the Research Institute of the McGill University Health Centre. This database is easily accessible via a web browser and multi-modal, with the ability to capture genetics data, medical history, medical imaging, detailed assessments of cognition and motor function, and patient-derived outcomes, among other things.

Within LORIS, data entry forms, or instruments, were created using the “Instrument Builder” module. Using the workflow found in the Methods section, 90 LORIS instruments were created, 62 of which had scoring algorithms developed to aid in data processing.

Detailed phenotyping including family history, perinatal history, developmental history, clinical evolution, time to event (i.e. time to reaching specific disease milestones such as loss of independent ambulation, dependency to tube feeding, etc.), neurological examination, neuropsychological assessment, etc. were developed in conjunction with other parent- and patient-reported outcomes such as quality of life, disability, and stress. The resulting instruments are summarized in Table 1.

**Table 1:** Developed Instruments of the LORIS MyeliNeuroGene Database for Rare Diseases

Instrument	Purpose
Family history	Inheritance pattern
Perinatal history	Disease onset/progression
Developmental history	Disease onset/progression
Investigations	Diagnostic Odyssey
Demographics	Sociodemographic variables
Clinical presentation	Disease onset/progression
Primary diagnosis	Disease onset/progression
Gross motor function measure—88	Measure changes in motor function
Leiter-3 intelligence scale	Measure changes in intelligence
Neuropsychological examinations	Measure changes in cognition
Rehabilitation	PT, OT, SLT, etc. used
Clinical evolution	Disease onset/progression
Time to event	Disease milestones
Clinical examination	Disease onset/progression
Swallowing Studies	VFSS and FEES evaluations
MRI analyses	Disease onset/progression
Modified Ashworth Scale (MAS)	Measure changes in spasticity
Fahn Marsden Scale (F-M)	Measure changes in dystonia
Global Dystonia Scale (GDS)	Measure changes in dystonia
Guy's Neurological Disability Scale (GNDS)	Measure disability and ADL
Gross Motor Function Classification System (GMFCS)	Characterize gross motor function
Communication Function Classification System (CFCS)	Characterize communication function
Manual Ability Classification System (MACS)	Characterize fine motor function
Eating and Drinking Ability Classification System (EDACS)	Characterize eating and swallowing functions
Scale for the Assessment and Rating of Ataxia (SARA)	Measure changes in ataxia
Non-communicating Children's Pain Checklist—Revised	Measure parent reported pain
Parent Reported Stress Questionnaires	Measure parental stress
Health-Related Quality of Life Questionnaires	Measure patient’s quality of life

*PT* physical therapy, *OT* occupational therapy, *SLT* Speech and language therapy, *VFSS* Video fluoroscopic swallow study, *FEES* Fiberoptic endoscopic evaluation of swallowing, *MRI* Magnetic resonance imaging, *ADL* Activities of daily living

One thousand patients and family members with rare diseases have been included into LORIS and assigned unique identifiers. This includes activation of enrollment, informed consent designation, external identifier logging, and family relationship mapping.

**Fig. 1:**
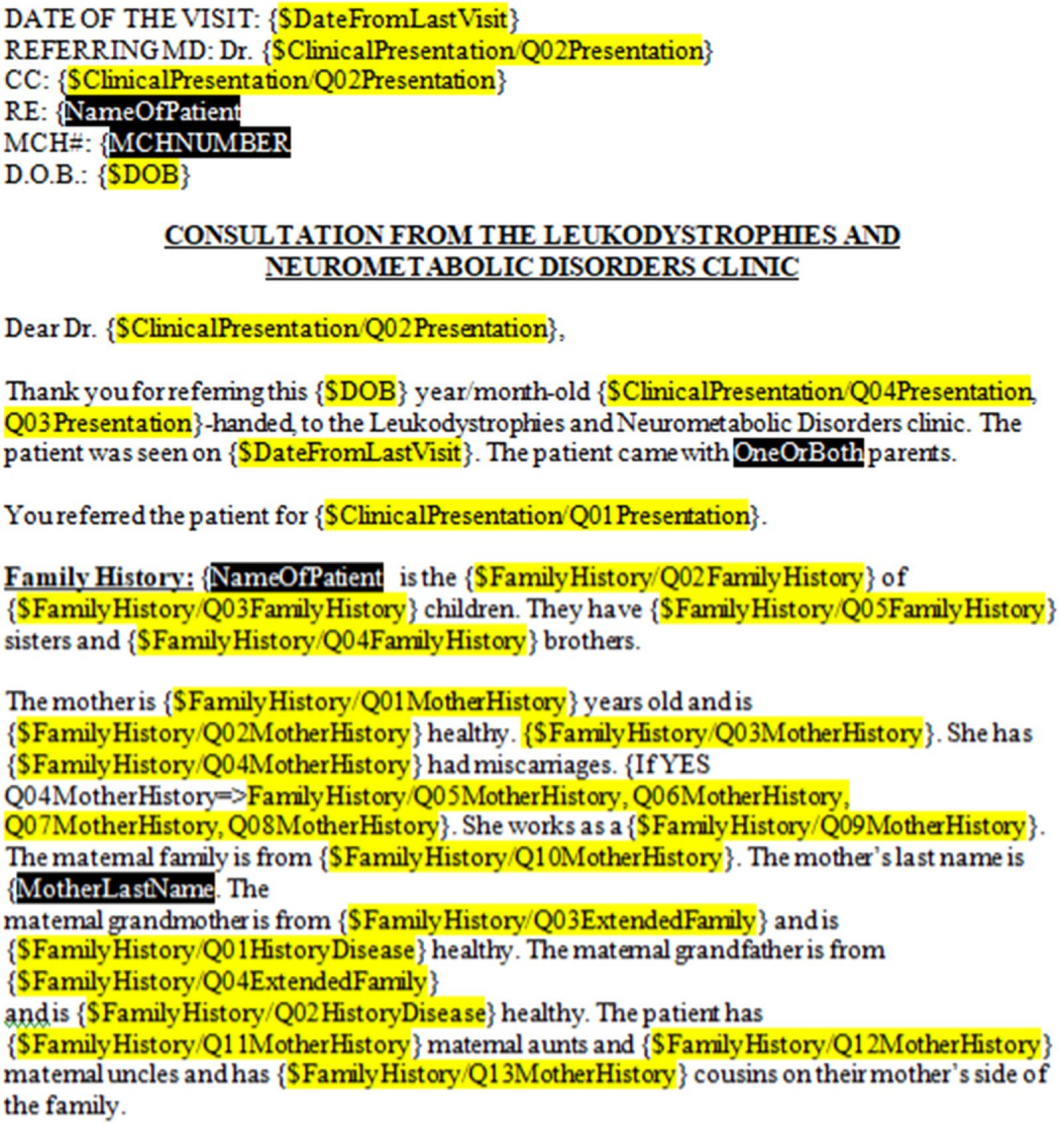
Screenshot of the LORIS MyeliNeuroGene dynamic letter generator: Yellow highlights customizable variables for the clinical letter generator. Black highlighted variables represent information that is not stored in LORIS and must be filled in by the physician

In addition, a dynamic letter generator is currently in development to assist in forwarding patient information to other physicians. The tool compiles the patient’s data, entered via the phenotyping instruments, into a Clinical Examination Letter. In place of the database field names, highlighted in yellow in Fig. 1, an instance of the letter renders the patient data for the corresponding field. The Clinical Examination Letter can be exported as an editable word document that details patient information, such as family history, clinical evolution, time to event and future plans for investigations. This letter can then be sent to the referring physicians for continuity of care, and has the advantage of not duplicating work done by the data entry clinician; as the clinician sees the patient and enters the data in the LORIS MyeliNeuroGene Database, the clinical note is auto-populated.

## Discussion

Most patients affected with rare diseases, from mildly to severely affected, support data sharing to promote research, healthcare, and knowledge transfer [18]. We have built and customized a LORIS database and detailed our workflow to aid rare disease researchers to create their own information management system, electronic health records, or database. There is a major need and benefit to sharing data in rare disease research. De-identifying and sharing information allows rare disease researchers to efficiently study disorders by collaborating and minimizing redundant studies [31], and by maximizing sample sizes.

An exportable dynamic letter generator has also been developed to save time when examining patients referred to the clinic. Patients with a rare disease who come to the Montreal Children’s Hospital undergo a battery of tests that can take up to two days to complete. These tests are performed in a standardized order at each visit (i.e. the order they appear in the database), to ensure consistency between research visits and research patients. All information is stored in the LORIS MyeliNeuroGene Database and can be exported in the form of a Clinical Examination Letter detailing all results, impressions, and plans to help treat the patients. This letter is then sent back to the referring physician for continuity of care. When this letter is written by hand it takes a few hours and introduces numerous chances for human error. Exporting the letter from quality-controlled instruments reduces this error and saves researchers’ and physicians’ time.

In addition to the clinical phenotyping instruments and dynamic letter generator, we have outlined, for the first time, the methodology to become Title 21 Code of Federal Regulations Part 11 Compliant, which is a requirement to use electronic records as historical controls in clinical trials in the United States [32, 33]. To our knowledge, our manuscript is the first to outline the requirements to adhere to 21 Code of Federal Regulations Part 11 Compliance. Future work will leverage the tools developed in this project to delineate the natural history of several rare diseases and will hopefully be used by clinicians and researchers around the globe.

## Conclusions

A major obstacle in rare disease research is overcoming small cohorts. Developing an online database that international collaborators can access and contribute to from all over the world is invaluable for increasing cohort sizes, discerning surrogate markers, and improving natural history data. Using this FDA compliant natural history data to validate outcome measures will be life-changing for patients and families because it will lead to historical control data that can be used in emerging clinical trials.

## Methods

### Title 21 code of federal regulations part 11 compliance (part 11 compliance) [34]

To adhere to Part 11 Compliance regulations, the LORIS MyeliNeuroGene Database has been customized to include additional security measures such as time stamped audit trails. We are currently implementing the electronic signatures and the 2-factor authentication. There is a gap in scientific literature detailing workflow and database development. As such, we will summarize the general requirements of Part 11 Compliance below and how they were implemented into our database.

#### Training verification

Users are required to have their credentials (e.g. education, training, experience) verified before performing tasks within the database. Written policy must be signed holding users accountable and responsible for their electronic signatures (discussed further below). This written policy must be stored, and a hard copy sent to the Office of Regional Operations (HFC-100), 5600 Fishers Lane, Rockville, MD 20857.

#### Biometrics

This is a method of verifying an individual’s identity based on a measurement of the individual’s physical features (i.e. fingerprints, etc.) or repeatable action that are unique to that person. In our case, we chose to use a unique pin separate from an authorized user’s password for 2-factor authentication.

#### Closed system

The **MyeliNeuroGene** database is a closed environment, meaning that access to the system is controlled by the same people who are responsible for the content of the electronic records. This includes the researchers and principal investigator. Operational audits on the system are done on a routine basis. Time stamp audit trails are tracked for each authorized user to trace creation, modification, or deletion of any instrument, visit, or other electronic record. User access is hierarchical, meaning some users do not have full access to the database and may only have “read” or “write” access. The database also must ensure that no user has the same pin or password, and that pins and passwords are periodically checked and changed to prevent unauthorized use. If unauthorized use occurs, there are immediate system security notifications. Per Canadian predicate rules, records must be stored for 25 years after study completion. United States record retention rules require storage for a minimum of 10 years.

#### Quality control

Processing pipelines must ensure data fits specific parameters and types. This is discussed in depth under the “Methods” section “LORIS Database and Workflow”.

#### Electronic signature

This includes any combination of text, graphic, data, audio, or other information that is represented in digital form by the database. Electronic signatures must include printed names of the signers, dates and times, meanings (e.g. approval, creation, reviewing), and an internal audit trail. These signatures are legally binding. Authority checks are completed every month to ensure only authorized users may sign, input, output, or modify records.

#### Digital signature

A digital signature combines the electronic signature and its corresponding cryptographic authentification, usually a pin and/or password that is used to verify the identity of the signer. It cannot be copied or pasted to or from another document, making it inexorably linked to the signed document. To not become cumbersome, continuous signing periods only require the first to be two factors authenticated with a biometric identification and password.

#### External auditing

It is highly recommended that after database development a third-party auditor inspects the system and documentation put in place. Auditors alert parties of any gaps or shortcomings and can advise developers of what needs to be changed for full compliance with local and international regulations. This will be organized for the MyeliNeuroGene database.

### LORIS database and workflow

#### Architecture

LORIS is a web-based data and project management software that stores demographic, clinical, behavioral, genetic, imaging, and patient-related outcomes accessible from any computer browser connected to the internet [27]. Multiple sites can enter, organize, and validate data under one management framework. Longitudinal data is organized around the “Subject Profile”. Clinical examination, imaging data, outcome measures, and metadata are organized by “Visits”. All stored information is de-identified and can be queried by an authorized user. Source documentation can be uploaded and affiliated with each visit. Quality control is ensured by automated scoring of clinical, behavioral and patient-reported outcomes, validating data types (string vs numerical), and requiring double data entry where necessary.

#### Workflow

**Fig. 2:**
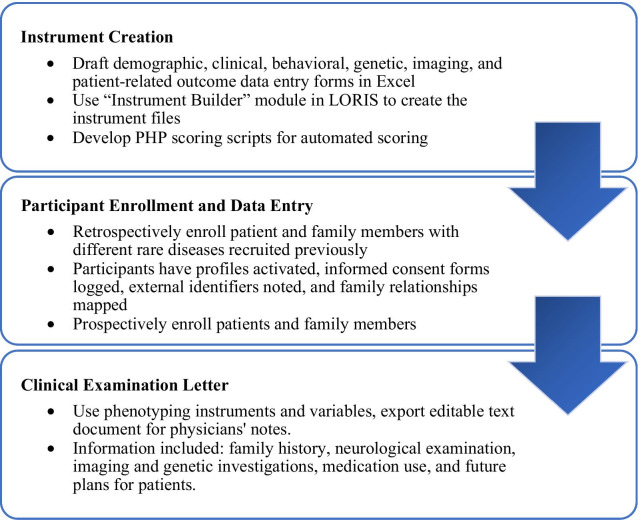
Database development workflow to create instruments, scoring algorithms, enroll patients, enter data, and output information into a clinical examination letter

To properly set up our rare disease database, we first began by drafting a data dictionary in the form of an Excel sheet. This spreadsheet outlined all of the data entry forms, or instruments, that would be developed using the LORIS Instrument Builder module detailed below. After instrument creation, participant enrollment and data entry can begin, with query and dissemination details tackled later. An overview of the workflow can be found in Fig. 2.

#### Instrument builder

Within LORIS are different modules to help researchers with no computer science or programming experience. The Instrument Builder module aids in the creation of demographic, clinical phenotyping, behavioral, genetic, imaging, and patient-related outcome measures. Each instrument can be customized with specific information such as a “Header”, “Label”, and “Scored Field” that give the instrument title, background information, and automatically calculated scoring respectively.

Data entry can be standardized using a “Textbox”, “Text area”, “Dropdown”, “Multiselect”, “Date”, and “Numeric” question entry. Each question is assigned a variable name “Question Name”, for calculations and data querying, and “Question Text” which asks the pertinent question at hand. For Dropdown questions, instrument specific options can be added for every question.

#### Instrument creation

Instruments were first planned and drafted using Excel in the form of a Data Dictionary. Columns consisted of Question Names, type of question (e.g. Numeric, Dropdown, etc.), Question Text, Question Options (available choices), and Formulas (for later calculations). Each row represented one question. Using the Data Dictionary and the Instrument Builder module on LORIS, each instrument was created: demographic forms, clinical phenotyping (i.e. spasticity and dystonia measures, gross and fine motor, eating and drinking function, ataxia, intelligence, disability, swallowing evaluations etc.), behavioral, genetic, imaging (i.e. MRI analyses), and patient-related outcomes (i.e. health-related quality of life, parental stress, pain characterization, etc.). Instruments’ files were then uploaded onto the MyeliNeuroGene private repository on GitHub as Pull Requests for review.

#### Scoring algorithms

After instrument completion, a PHP scoring script was developed for instruments that required them. Automatic scoring reduces human error and dramatically decreases time spent on calculations. Scoring scripts were also uploaded onto the GitHub repository for review.

#### Instrument implementation

After instruments and scoring scripts were developed, they were uploaded to the MyeliNeuroGene private repository on GitHub as Pull Requests for review. After revision and modification (if necessary), the Pull Requests were approved, and the instruments made available on an insulated LORIS staging server where beta testing occurred. After testing was completed, instruments were pushed to the LORIS production server for instrument pipeline completion and data entry.

#### Participant enrollment

Before data entry could be completed, Subject Profiles had to be entered. Our group has consented more than 1000 patients and family members with different rare diseases since 2011, and patient and family recruitment is ongoing. To create a new profile, “Date of Birth”, “Sex”, “Site” (in the case of a multi-site study), and “Project” must be entered. Projects can be separated into different studies such as natural history, imaging, genetic, or even clinical trials assessing therapeutics. A new Subject Profile, or candidate, generates two identifier codes, a DCCID and a PSCID which are unique LORIS identifiers.

After the creation of the Subject Profile, each candidate was activated in the study, designated for which informed consent form was signed, and mapped to any external identifier codes. Under “Participant Status”, we tracked the participant’s status in the study (e.g. Active, Death, Lost to Follow-up, etc.). Comments can be entered with both time, date, and author history tracked in the internal audit trail. “Consent Status” tracks the latest signed Research Ethics Board (REB) approved informed consent form. Finally, mapping the “External Identifier” is crucial for future correspondence with family doctors and other collaborators.

#### Data entry

**Fig. 3:**
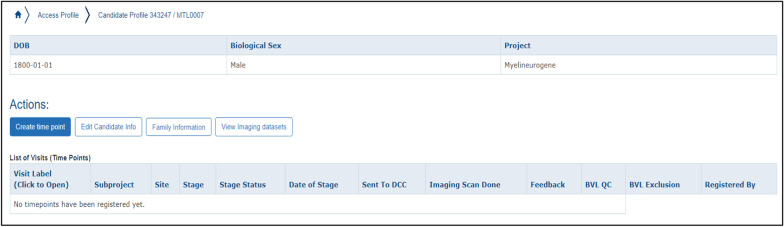
Creating longitudinal time points for patient visits

**Fig. 4:**
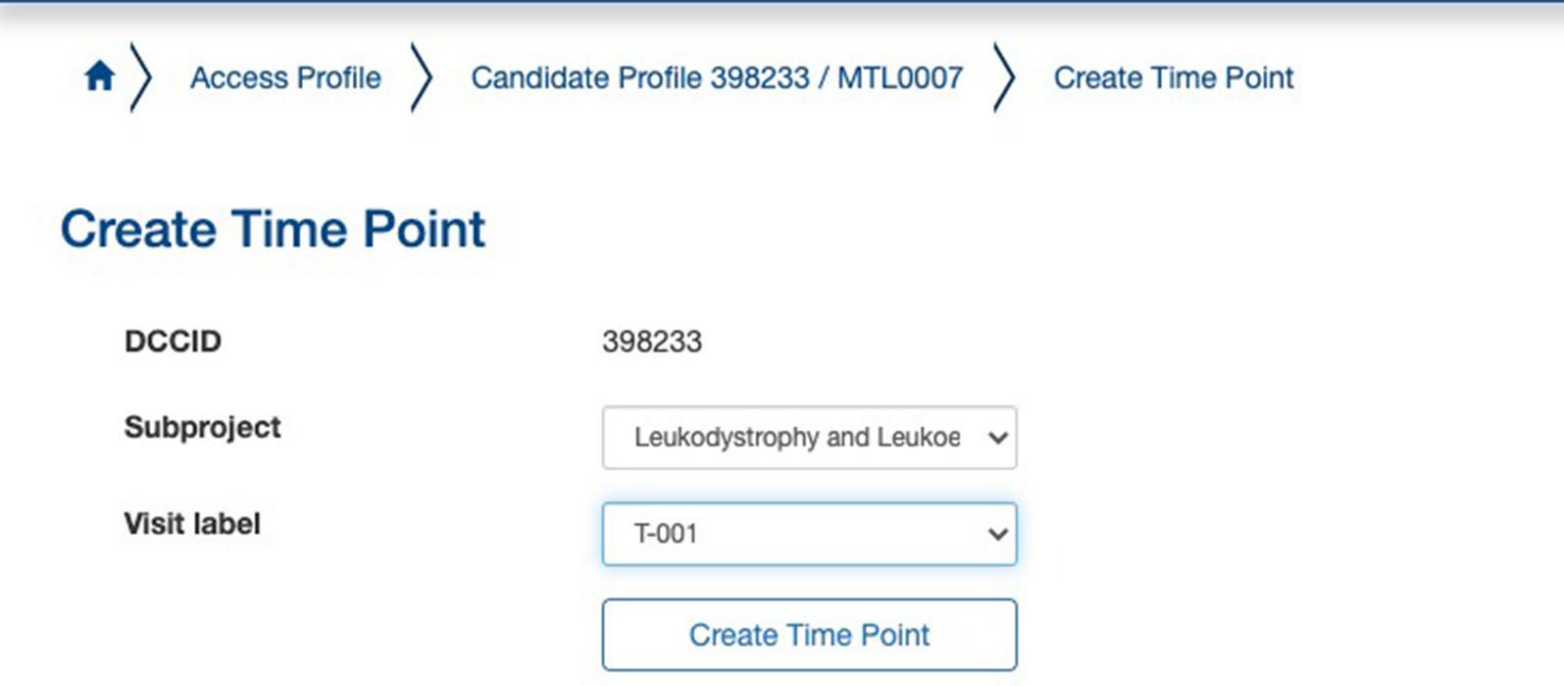
Associating time points with subprojects

**Fig. 5:**
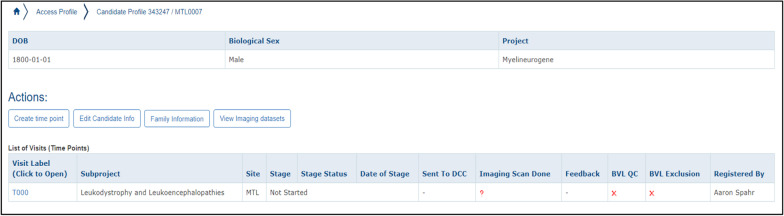
Visualizing time point information in the LORIS Candidate Profile

“Create time point” allows for data entry of clinical, behavioral, and patient determined outcomes that were created during the Instrument Creation process. It also enables uploading of any imaging data collected. We customized our time points to correspond to the age of the patient. For instance, a participant’s birth date would be time point T000, and a follow-up appointment 6 months later would be time point T006. A prenatal examination 1 month before a T000 examination would be designated as T-001. The steps to creating a time point can be seen in Figs. 3, 4, and 5.

**Fig. 6:**
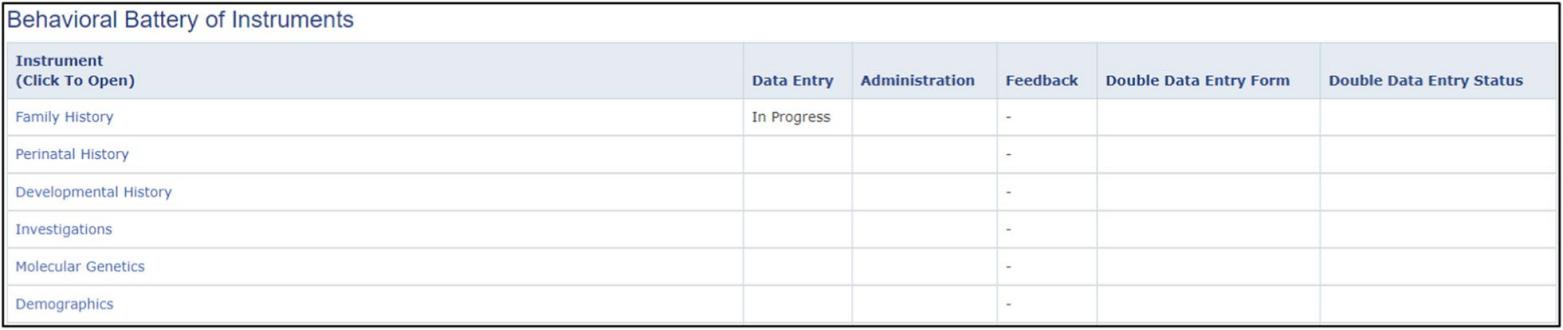
Test battery of instruments customized for each participant based on time point and age appropriateness

Selecting time point T000 opens a page for all instruments developed to work on our database (Fig. 6). Time points can be customized so that only specific instruments are available to participants at specific ages. Entering multiple visits allows for prospective tracking.

#### Family information

We have further customized LORIS to include Family Relationship information. Linking de-identified individuals allows us to link a given patient’s disease characteristics to his/her parents’ reported measures such as parental stress or patient/parents/sibling’s quality of life. It also allows us to organize family genetic results when next generation sequencing (NGS) investigations are being conducted as well as any family/parent reported outcomes.

## Data Availability

The datasets used and/or analyzed during the current study are available from the corresponding author on reasonable request.

## Abbreviations

ADL: Activities of Daily Living
CFCS: Communication Function Classification System
CFR: Code of Federal Regulations
DCCID: LORIS Identifier
EDACS: Eating and Drinking Ability Classification System
FDA: Food and Drug Administration
FEES: Fiberoptic Endoscopic Evaluation of Swallowing
GDS: Global Dystonia Scale
GMFCS: Gross Motor Function Classification System
GNDS: Guy's Neurological Disability Scale
HID: Human Clinical Imaging Database
LORIS: Longitudinal Online Research and Imaging System
LONI: LONI Image Data Archive
MACS: Manual Ability Classification System
MAS: Modified Ashworth Scale
MRI: Magnetic Resonance Imaging
NGS: Next Generation Sequencing
OT: Occupational Therapy
PHP: PHP: Hypertext Preprocessor
PSCID: LORIS Identifier
PT: Physical Therapyt
REB: Research Ethics Board
SLT: Speech and Language Therapy
VFSS: Video Fluoroscopic Swallow Study
